# Effect of tobacco smoking on the epigenetic age of human respiratory organs

**DOI:** 10.1186/s13148-019-0777-z

**Published:** 2019-12-04

**Authors:** Xiaohui Wu, Qingsheng Huang, Ruheena Javed, Jiayong Zhong, Huan Gao, Huiying Liang

**Affiliations:** 1 Institute of Pediatrics, Guangzhou Women and Children’s Medical Center, Guangzhou Medical University, No. 9 Jinsui Road, Guangzhou, 510623 Guangdong China; 2 Department of Medical Genetics, School of Basic Medical Sciences, Southern Medical University, Guangzhou, Guangdong China; 3 Guangdong Technology and Engineering Research Center for Molecular Diagnostics of Human Genetic Diseases, Guangzhou, Guangdong China; 4 Guangdong Province Key Laboratory of Psychiatric Disorders, Guangzhou, Guangdong China

**Keywords:** Smoking, Respiratory system, Aging, Epigenetic clocks, Methylation

## Abstract

**Background:**

Smoking leads to the aging of organs. However, no studies have been conducted to quantify the effect of smoking on the aging of respiratory organs and the aging-reversing ability of smoking cessation.

**Results:**

We collected genome-wide methylation datasets of buccal cells, airway cells, esophagus tissue, and lung tissue from non-smokers, smokers, and ex-smokers. We used the “epigenetic clock” method to quantify the epigenetic age acceleration in the four organs. The statistical analyses showed the following: (1) Smoking increased the epigenetic age of airway cells by an average of 4.9 years and lung tissue by 4.3 years. (2) After smoking ceased, the epigenetic age acceleration in airway cells (but not in lung tissue) slowed to a level that non-smokers had. (3) The epigenetic age acceleration in airway cells and lung tissue showed no gender difference.

**Conclusions:**

Smoking can accelerate the epigenetic age of human respiratory organs, but the effect varies among organs and can be reversed by smoking cessation. Our study provides a powerful incentive to reduce tobacco consumption autonomously.

## Introduction

Tobacco smoking accelerates the process of organ aging and leads to multiple diseases [1–3]. The respiratory system, the frontline attacked by toxic substances in tobacco, often falls victim to diseases such as chronic bronchitis, emphysema, and chronic obstructive pulmonary disease [4]. Various studies also suggest that smoking can trigger aging-related changes, from cell phenotype to gene expression and epigenetic regulation, in the respiratory system. Fortunately, smoking cessation can effectively reverse these changes [5]. A photonumeric scale has been developed to confirm that smokers’ perioral wrinkles are deeper than non-smokers [6]. Smoking dysregulates 18 age-related genes and shortened telomere length in small airway epithelia [2]. Cigarette-smoke exposure induces autophagy impairment, producing aggresome bodies that accelerate lung aging [7]. Current-smokers, compared to never-smokers, are linked to a 0.74–2.4% decrease of DNA methylation in 11 loci located in lung cancer-related genes [8]. Smoking cessation relieves respiratory symptoms and bronchial hyperresponsiveness and prevents an excessive decline in lung function [9]. Peripheral blood epigenetic data are used to confirm that the effects of smoking on DNA methylation are partially reversible 3 months after smoking cessation [10]. Most of these studies, however, have described the age-related changes qualitatively but not quantitatively, and few have tested the self-healing ability of organs after smoking cessation.

Recent studies have quantified the aging process in the tissue by DNA methylation spectroscopy [11–13], finding that tobacco smoking is associated with epigenetic alterations [13, 14]. However, most of these epigenetic biomarkers of aging are developed using data from whole blood. Although they can be applied to other tissue samples, the ability of prediction is unstable [11, 13]. Fortunately, the “epigenetic clock” method, developed by 51 different tissues and cell types, has effectively solved this problem [12]. Simultaneously, it overcomes the technical obstacles in measuring telomere length in various human systems, including the respiratory system [12]. At present, the ability of epigenetic clock to predict aging has been verified in studies concerning diet [15], obesity [16], lifetime stress [17], centenarian status [18], Down syndrome [19], osteoarthritis [20], Alzheimer’s disease [21], and Parkinson’s disease [18], but we cannot find analysis on smoking-induced epigenetic age acceleration in the respiratory system.

Here, with the “epigenetic clock” method proposed by Horvath [12], we evaluated the epigenetic age of buccal cells, airway cells, esophagus, and lung tissue in non-smokers, smokers, and ex-smokers. We quantified the age acceleration by smoking and the aging-inhibiting effect of smoking cessation. The acceleration of epigenetic age may be used to evaluate the detrimental effects of smoking on the respiratory system. Our finding may be encouraging for smokers to cease smoking.

## Results

### Epigenetic age of respiratory system measured by DNA methylation

**Fig. 1:**
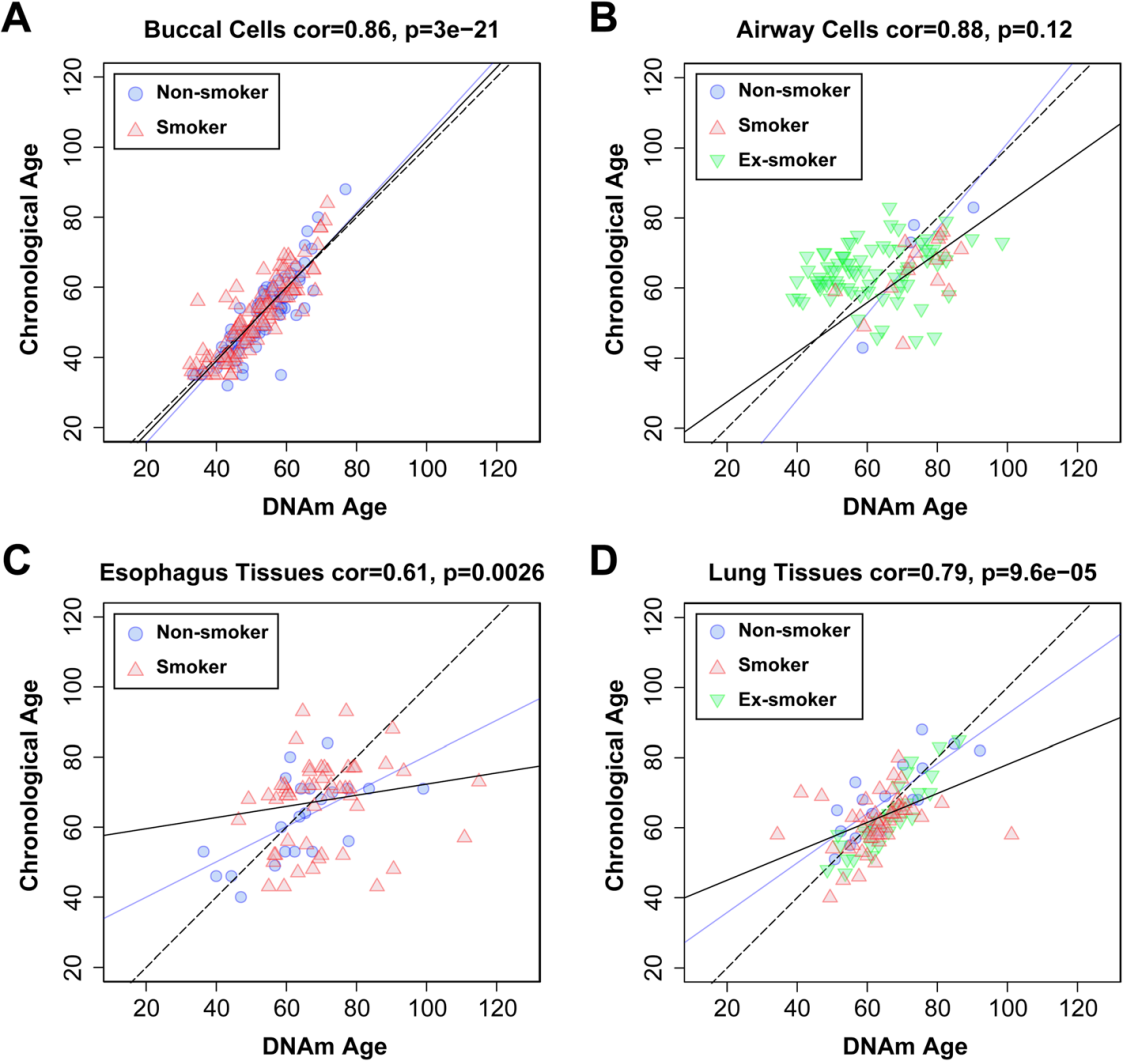
Correlation between epigenetic age and chronological age. The figure shows scatter plots of epigenetic age (*x*-axis) against chronological age (*y*-axis) in buccal cells (**a**), airway cells (**b**), esophagus tissue (**c**), and lung tissue (**d**). Blue circles, red triangles, and green inverted triangles indicate non-smokers, smokers, and ex-smokers, respectively. The dashed line is the diagonal where epigenetic age equals to chronological age, the blue solid line is the regression of epigenetic age on chronological age for non-smokers, and the black solid line is the regression of epigenetic age on chronological age for all the smokers and non-smokers

We found that the DNAm age of non-smokers had a strong linear relationship with the chronological age of the major organs in the respiratory system except the airway cells (Fig. 1). The null hypothesis of uncorrelation for the airway cells cannot be rejected due to the small sample size (*p* = 0.12, only four data points, Fig. 1b). We will address the problem of insufficient sample size later in this study. The linear correlation relationship also holds if merging together the smokers and non-smokers (Fig. 1, black lines). From all the predictions, we found that the DNAm age of airway cells and lung tissue in smokers tended to be greater than that of non-smokers.

### Smoking accelerates aging of airway cells and lung tissue

**Fig. 2:**
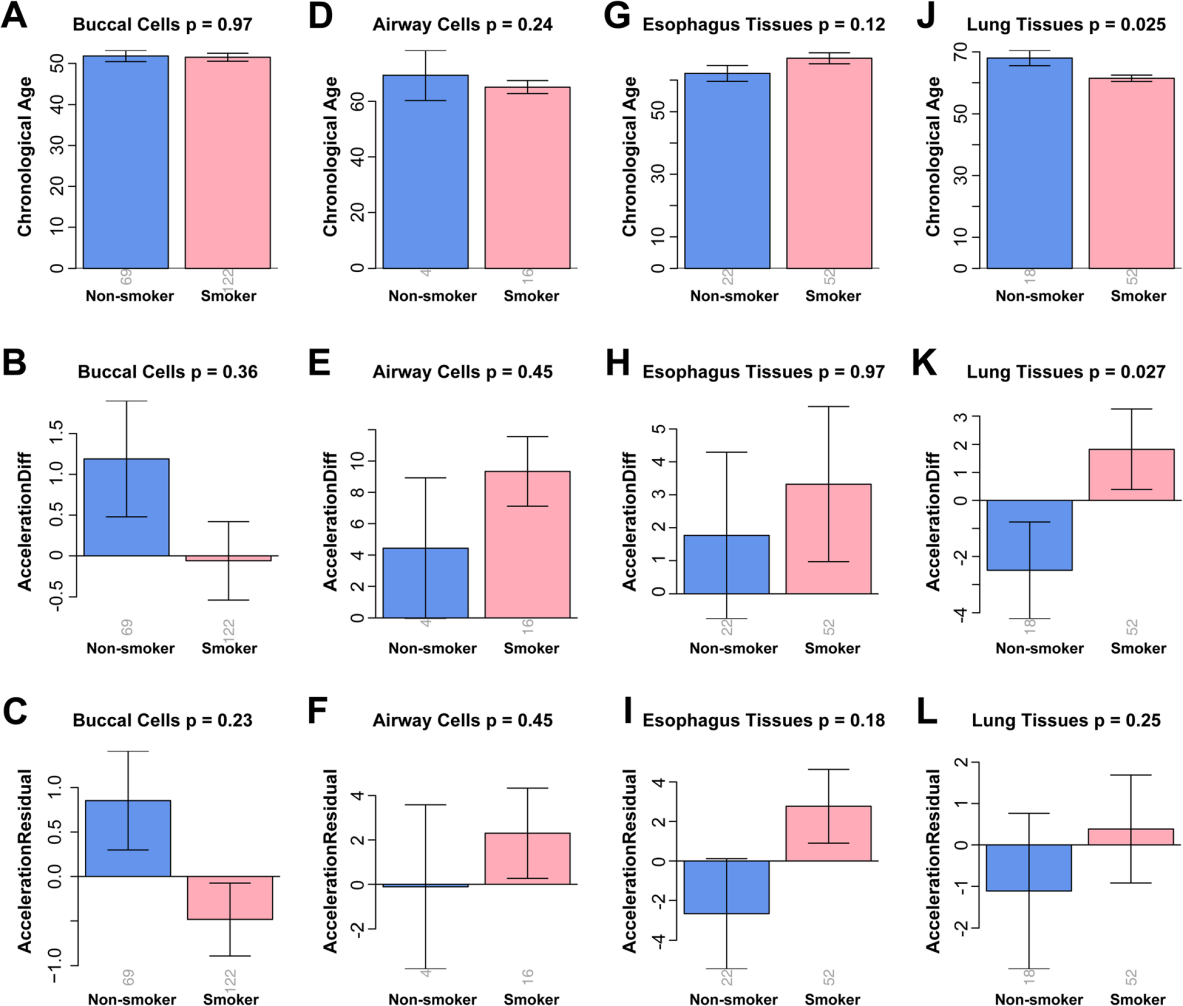
Comparison of chronological age and epigenetic age acceleration between smokers and non-smokers. Columns correspond to buccal cells (**a**–**c**), airway cells (**d**–**f**), esophagus tissue (**g**–**i**), and lung tissue (**j**–**l**). Rows correspond to the chronological age (**a**, **d**, **g**, and **j**), AccelerationDiff value (**b**, **e**, **h**, and **k**), and AccelerationResidual value (**c**, **f**, **i**, and **l**). The latter two measure epigenetic age acceleration. The *p* value from Kruskal-Wallis tests is written above each sub-figure

We conducted Kruskal-Wallis tests to verify whether smoking accelerates aging in airway cells and lung tissue, via two measurements on age acceleration developed by Horvath [12]. The first is AccelerationDiff which equals to DNAm age minus chronological age; the second is AccelerationResidual based on the residual generated by the linear regression (Fig. 1, the black solid lines) of DNAm age on chronological age [12]. A positive (negative) value of age acceleration suggests that the organ is older (younger) than its chronological age. In the lung tissue, the AccelerationDiff value of smokers exceeded that of non-smokers by 4.3 years (*p* = 2.7 × 10^−2^, Fig. 2k). In the airway cells, the AccelerationDiff value of smokers exceeded that of non-smokers by 4.9 years, but the difference was not significant (*p* = 0.45, Fig. 2e), probably due to the small sample size. In the buccal cells, the AccelerationDiff value of smokers was insignificantly lower than that of non-smokers by 1.64 years (*p* = 0.36, Fig. 2b). In the esophagus tissues, the AccelerationDiff of smokers was insignificantly higher than that of non-smokers, only by 0.99 years (*p* = 0.97, Fig. 2h). All the AccelerationResidual values did not show significant differences between smokers and non-smokers (buccal cells, *p* = 0.23; airway cells, *p* = 0.45; esophagus tissues, *p* = 0.18; lung tissue, *p* = 0.25; Fig. 2c, f, i, and l), but these differences showed trends similar to those in AccelerationDiff value. For example, age acceleration of smokers was faster than that of non-smokers in the airway cells, esophagus tissue, and lung tissue. The difference of chronological age between the two groups was minor in buccal cells, airway cells, and esophagus tissues (buccal cells, *p* = 0.97; airway cells, *p* = 0.24; esophagus tissues, *p* = 0.12; Fig. 2a, d, and g). In lung tissues, the chronological age of non-smokers was significantly greater than that of smokers (lung tissue, *p* = 2.5 × 10^−2^, Fig. 2j). Thus, smoking can accelerate the aging of the lung and probably also the airway.

### Smoking cessation alleviates epigenetic aging in airway cells but not in lung tissue

**Fig. 3:**
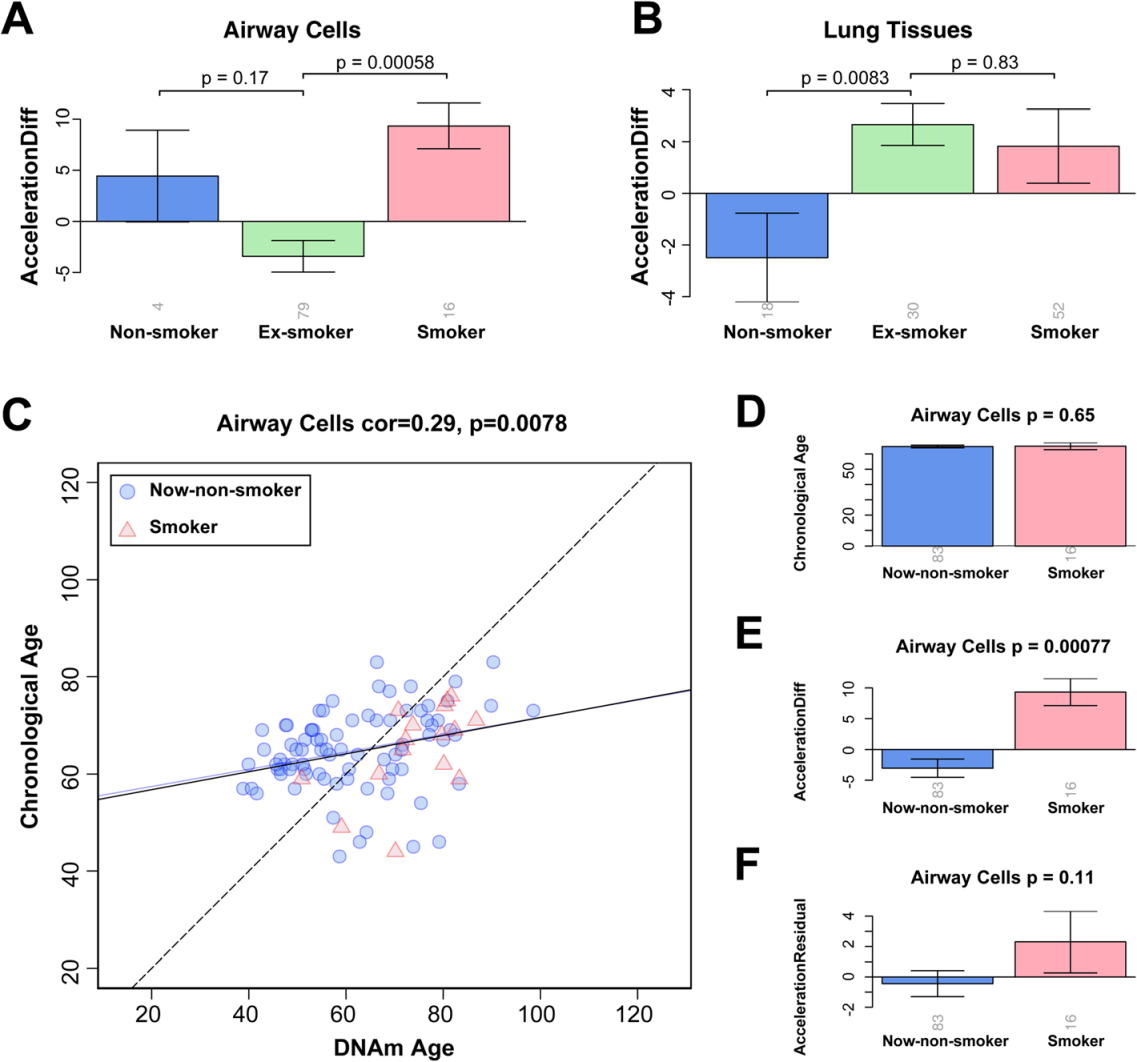
Change in epigenetic age acceleration after smoking cessation in airway cells, lung tissue and merged (for airway cells) groups. **a**, **b** Comparison of epigenetic age acceleration between non-smokers, smokers, and ex-smokers in airway cells (**a**) and lung tissue (**b**). **c** A scatter plot of epigenetic age (*x*-axis) against chronological age (*y*-axis) in the airway cells (Fig. 1b). Blue circles indicate now-non-smokers (merge of non-smokers and ex-smokers) and red triangles indicate smokers. The dashed line is the diagonal where epigenetic age equals to chronological age, the blue solid line is the regression of DNAm age on chronological age for now-non-smokers, and the black solid line is the regression of DNAm age on chronological age for all of smokers and now-non-smokers. **d**–**f** The comparison of chronological age (**d**), AccelerationDiff value (**e**), and AccelerationResidual value (**f**) between smokers and now-non-smokers, as updates of Fig. 2d–f, where ex-smokers were not included in analysis

In the airway cells, we found that smoking cessation effectively lowered the rate of aging (ex-smokers vs. smokers, *p* = 5.8 × 10^−4^; Fig. 3a) down to that of non-smokers (ex-smokers vs. non-smokers: *p* = 0.17, Fig. 3a). However, in the lung tissues, the accelerated aging rate did not fall significantly after smoking cessation (ex-smokers vs. smokers, *p* = 0.83; ex-smokers vs. non-smokers, *p* = 8.3 × 10^−3^; Fig. 3b), suggesting that smoking damage to the lung was permanent and irreversible.

For the airway cells, the AccelerationDiff value of smokers was larger than that of non-smokers, but the difference was not significant, probably because of the small sample size of non-smokers. Given that there was no significant difference in AccelerationDiff value between ex-smokers and non-smokers, we merged these two groups into a “now-non-smoker” group and re-tested whether smoking accelerated epigenetic aging of airway cells. The results showed that DNAm age of airway cells in now-non-smokers had a strong linear relationship with chronological age (Fig. 3c). The new comparison showed that in the airway cells, the aging rate of smokers was significantly higher than that of now-non-smokers (*p* = 7.7 × 10^−4^ for AccelerationDiff and *p* = 0.11 for AccelerationResidual, Fig. 3e, f). Also there was no significant difference in the chronological age between the two groups (*p* = 0.65, Fig. 3d).

### Smoking cessation restores the methylation level of airway cells but not lung tissue

**Fig. 4:**
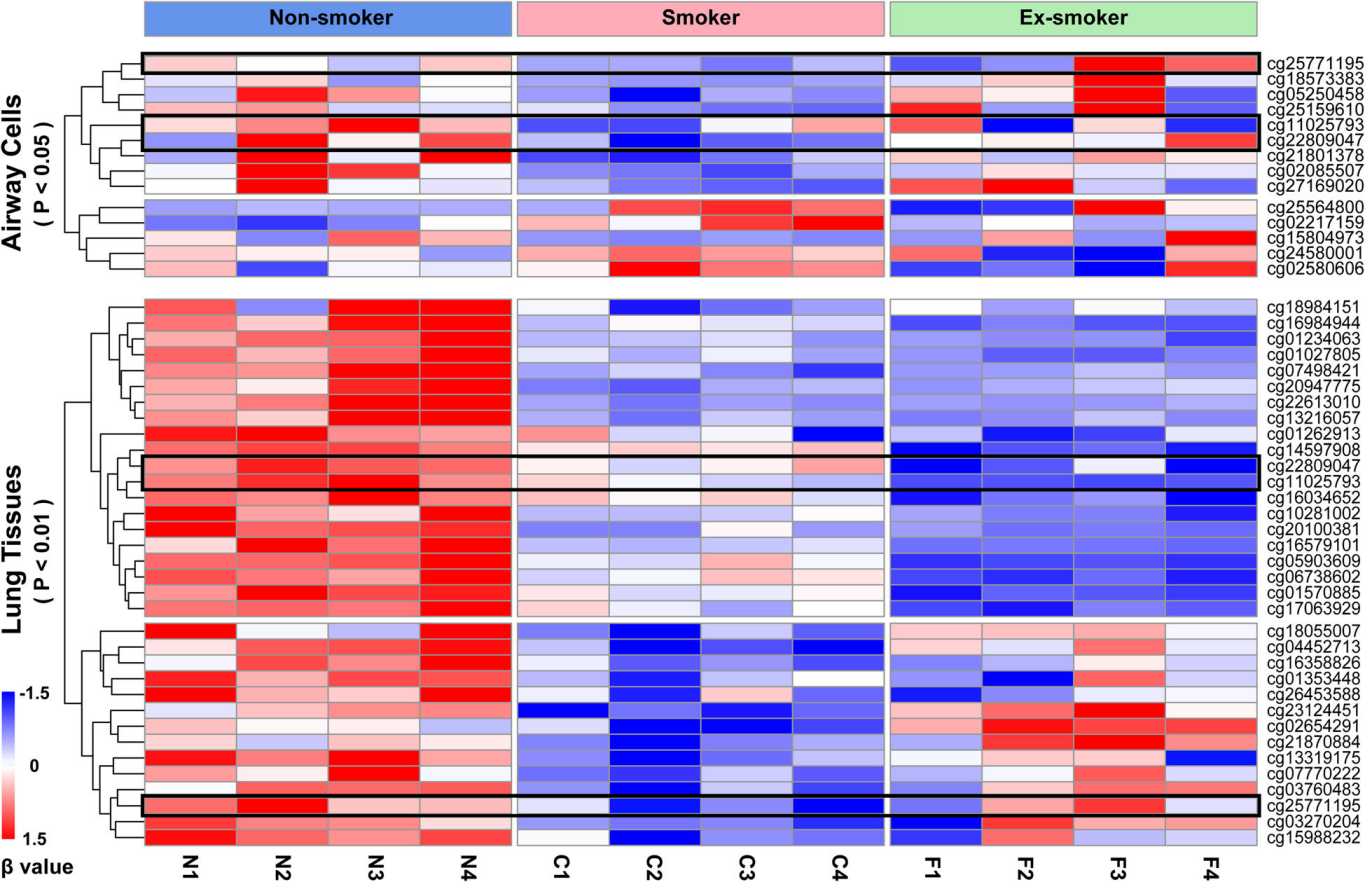
DNA methylation levels in non-smokers, smokers, and ex-smokers. The color scales indicate normalized DNA methylation levels, *β* value. The upper part of the heat map shows gene site clusters in airway cell samples, filtered by a methylation level difference (*p* < 0.05) between sites; the lower part, in lung tissue samples, filtered by a methylation level difference (*p* < 0.01) between sites. Three sites shared by airway cells and lung tissue are marked with black boxes

Based on the 353 CpGs of epigenetic clock, we randomly selected four samples of airway cells and lung tissue from each non-smoker, smoker, and ex-smoker groups (24 samples in total). Firstly, we performed differential methylation analysis on the airway cells and lung tissue of non-smokers and smokers. Fourteen differential methylation sites were screened out in airway cells (*p* < 0.05) and 34 in the lung tissue (*p* < 0.01). Subsequently, the above-selected sites were extracted from airway cell samples and lung tissue samples of ex-smokers, and the methylation levels at these sites were observed. We found that the methylation levels of 14 sites in airway cell samples from ex-smokers changed remarkably compared with those of smokers, and tended to return to a level that non-smokers had. However, most sites showed no significant decrease of methylation level in lung tissue samples from ex-smokers (Fig. 4).

By GO analysis, we found that the functions of these sites were enriched in cell senescence and apoptosis, regulation of metabolic processes, tissue development, and Alzheimer’s disease. The reduction in methylation level at these sites can be used as an index for smoking-induced accelerated aging.

### Gender had no effect on smoking-induced epigenetic age acceleration

**Fig. 5:**
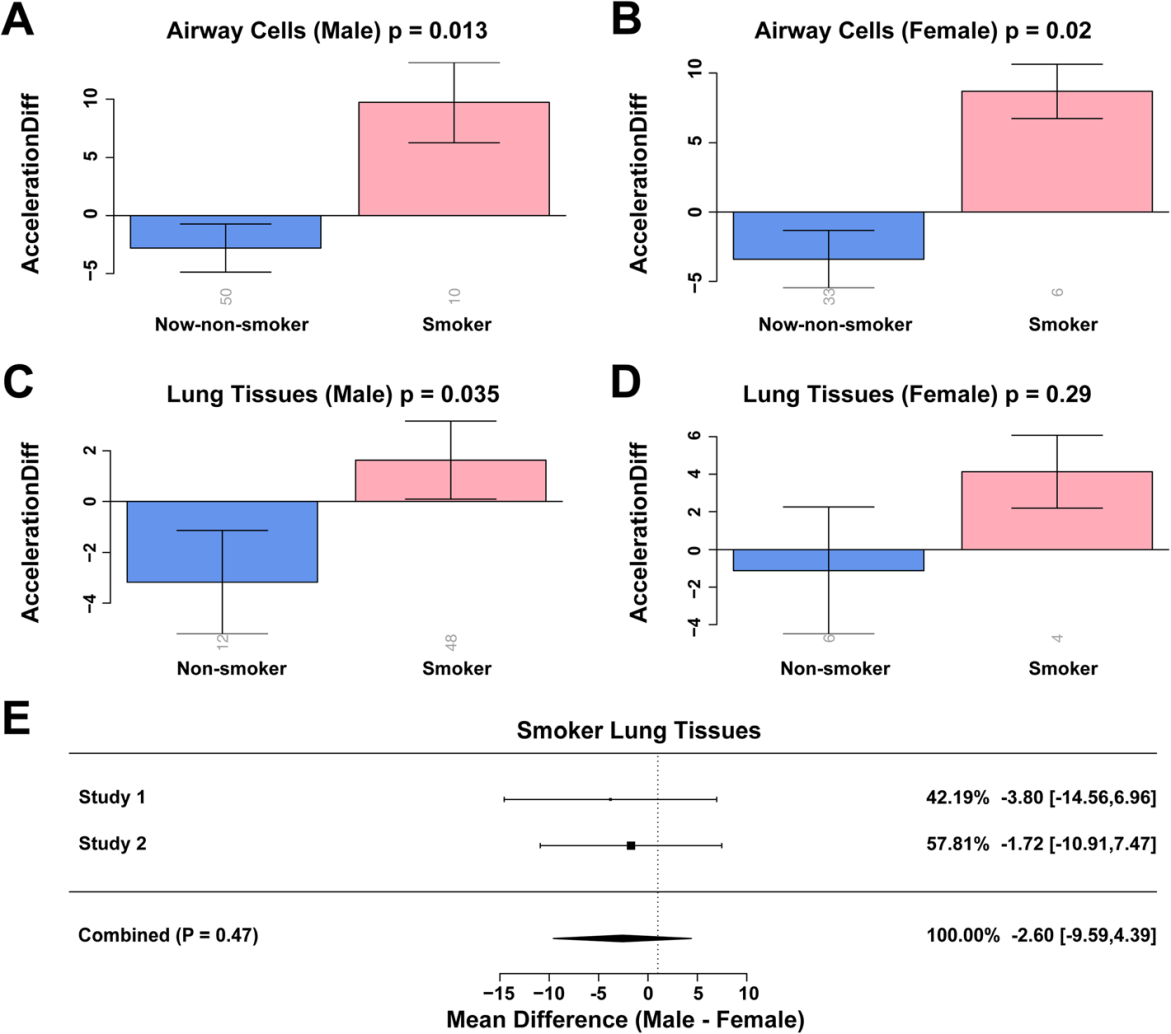
Comparison of epigenetic age acceleration between smokers and non-smokers grouped by gender. Comparison of epigenetic age acceleration of airway cells (**a**, **b**) and lung tissue (**c**, **d**) between smokers and non-smokers, grouped by gender (males, **a** and **c**) and (females, **b** and **d**). **e** A forest plot generated by the meta-analysis on the gender effect on epigenetic age-acceleration of lung tissue in smokers. Each row in the forest plot shows the mean difference in epigenetic age acceleration between males and females in smokers’ lung tissue and the 95% confidence interval

We found that in the airway cells of both men and women, smoking accelerated the rate of epigenetic aging (*p* = 1.3 × 10^−2^ for males and *p* = 2.0 × 10^−2^ for females, Fig. 5a, b). But in the lung tissue, only men showed significantly accelerated aging (*p* = 3.5 × 10^−2^ for males and *p* = 0.29 for females, Fig. 5c, d). To eliminate the bias introduced by the small-size female tissue sample, we used meta-analysis to explore two independent lung tissue datasets of male and female smokers. The meta-analysis suggested that the lung tissue of male smokers did not age faster than that of female smokers (*p* = 0.47, Fig. 5e).

## Discussion

Our study quantified the effect of smoking on the aging of respiratory organs based on the “epigenetic clock” method. Our main findings were (1) smoking significantly accelerated the aging of airway cells and lung tissue, but not buccal cells and esophagus tissue; (2) after smoking cessation, the accelerated aging was slowed in the airway cells, but not in the lung tissue; and (3) gender brought no difference in the epigenetic age acceleration in airway cells and lung tissue. We had also tried other estimate methods (developed by Hannum et al. and Levine et al., respectively) [11, 13]; however, the prediction accuracy was not satisfactory. This is probably because they were developed from whole blood datasets, yet the “epigenetic clock” was developed from the multi-tissue datasets [12] which was more suitable for this study.

Our study found that the methylation levels in the airway cells and lung tissue changed, a possible reason for altered gene expression detected in previous studies. Differentially expressed genes have been analyzed in small airway epithelia between healthy non-smokers and healthy smokers [2]. Among them, TRIP10 is a multi-domain adaptor protein whose differential DNA methylation can promote cell survival or cell death in a cell-type-dependent manner [22]. We found that the methylation level of cg02085507 located upstream of TRIP10 gene was significantly reduced by smoking, corresponding to the upregulation of this gene in Matthew’s study [2]. RPL31 gene associated with cg22809047 is the optimal combination member of ECFCs senescence housekeeping genes [23]. This site is a differential methylation site in the airway cells and lung tissue, but only the methylation level in airway cells rose to the normal after smoking cessation. In addition, cg15804973 in airway cells is linked to the longevity-associated MAP3K5 gene [24]. Cg07498421 found in lung tissue is associated with CRADD (also known as RAIDD), an adaptor protein containing a death domain that can oligomerize with PIDD and caspase-2 to initiate apoptosis [25]. Furthermore, Yafang et al. identified differentially expressed genes between non-smokers and smokers [26], among which the methylation sites related to CBX7, DKK3, TBX5, SCD5, PTGER2, and BIK genes also showed differential methylation levels in our study. These genes are involved in age-related functions such as cellular senescence [27], skeletal muscle atrophy [28], cardiac malformations [29], brain neurotrophic factors [30], hair follicle growth [31], and HSC hematopoietic function [32]. These studies strongly support our argument that smoking can accelerate the aging of airway cells and lung tissue.

Smoking accelerates the epigenetic age in manners varying among respiratory organs. We surmise that the difference results from the exposure dosage, which is determined by the duration of being exposed to, the surface area contacting to, and the retention capacity of toxins from cigarettes. On the one hand, smoking did not significantly accelerate the aging of buccal cells and esophagus tissue. The buccal cells contact with the toxins transiently though it bears the first brunt. The esophagus tissue is exposed to toxins longer, but with a narrow exposed area. On the other hand, smoking accelerates the aging of airway cells and lung tissue (faster in the later), because the toxins travel a long time through the airway and eventually anchor at the lung.

Smoking cessation at any time can benefit the prevention and treatment of respiratory diseases [33]. The role of smoking cessation in improving airway and lung function is unquestionable, but its epigenetic mechanism is unclear. We found that after smoking cessation, the methylation level was better reversed in the airway than in the lung, which confirms previous studies on the recovery effect of smoking cessation. For example, the peripheral blood also has the ability of reversible methylation states after smoking cessation [10, 13, 34].

## Conclusion

We concluded that smoking can accelerate the epigenetic aging of human respiratory organs, but this effect varies among organs and can be reversed by smoking cessation.

## Materials and methods

### DNA methylation datasets

We collated genome-wide methylation datasets of buccal cells, airway cells, esophagus tissue, and lung tissue. These datasets contained records of tobacco exposure (Additional file 1: Table S1). Briefly, dataset 1 was the epigenome-wide association study based on oral rinse samples from a case-control study of 154 cases and 72 controls [35]; dataset 2 presented the global methylation data of buccal cells collected from non-smokers, smokers and moist snuff consumers (40 subjects/cohort) [36]; dataset 3 consisted of small airway cells from 38 ex-smokers: 15 subjects with COPD and 21 with normal lung functio n[37]; dataset 4 analyzed the genome-wide DNA methylation of airway cells from individuals with and without COPD (non-COPD *n* = 26, COPD *n* = 35) [38]; dataset 5 evaluated the genome-wide methylation levels in 81 esophageal tissues using HumanMethylation450 Bead Chips (Illumina), including BE, dysplastic BE and EAC epithelial cell s[39]; dataset 6 covered lung tissues of patients including eight lifelong non-smokers, eight current smokers, and eight patients with COPD [40]; dataset 7 included tumor and adjacent non-tumor tissues excised from a cohort of 35 patients with stage I lung adenocarcinoma [41]; dataset 8 contained 24 samples of freshly extracted and frozen tissues from early adenocarcinoma. Smokers/non-smoker group included 6 cases with paired tumor and non-tumor tissue [42]; dataset 9 included 28 fresh-frozen lung cancer samples, 28 microscopically normal lung tissues, and 6 additional non-tumor control lung tissues. They were all analyzed in parallel by bead array methylation [43]. All of the datasets were measured on the Illumina 450 K array except two measured on the Illumina 27 K array.

### Determination of epigenetic age acceleration

The normal samples (non-tumor) in the above datasets were selected, and their DNA methylation ages were measured using the epigenetic clock [12]. The epigenetic clock was defined as an age-predicting method based on the DNA methylation level at 353 CpG sites. We referred to the predicted age as the DNAm age. The universal measure of age acceleration (AccelerationDiff) was defined as the difference between the DNAm age and the chronological age; another measure of acceleration (AccelerationResidual) equaled the residual resulting from linear regressing DNAm age on chronological age (Fig. 1, black lines). Epigenetic clock procedures were performed according to the instructions on the website (https://dnamage.genetics.ucla.edu) [12].

### Statistical analysis between groups

The Kruskal-Wallis test was applied to determine the significant difference between the two groups (non-smokers/smokers or non-smokers/ex-smokers or ex-smokers/smokers). The effect of gender in smokers on the methylation age of lung tissue was determined by meta-analysis. *p* values of less than 0.05 were considered to be significant. Statistical analysis was performed using R Studio. The meta-analysis was implemented in the *metafor* R package; the forest plot was drawn by the *forestplot* R package.

### Differential methylation analysis between three smoking states

The differences in methylation levels at 353 age-related methylation loci between non-smokers and smokers groups were examined using the *limma* package in R Studio. The *heatmap* was drawn by the *pheatmap* R package.

## Supplementary information

**Additional file 1: Table S1.** Summary of the DNA methylation datasets.

## Data Availability

The 27 k (GSE55454, GSE63384) and 450 k (GSE70977, GSE94876, GSE111396, GSE89181, GSE92511, GSE83842, GSE94785) methylation datasets analyzed during this study are available in the NCBI GEO (Gene Expression Omnibus) repository, https://www.ncbi.nlm.nih.gov/geo/.

## Abbreviations

DNA: Deoxyribonucleic acid
GO: Gene Ontology
ECFCs: Endothelial colony forming cells
CRADD (RAIDD): CASP2 and RIPK1 domain containing adaptor with death domain
PIDD: p53-induced death domain protein 1
HSC: Hematopoietic stem cell
COPD: Chronic obstructive pulmonary disease
BE: Barrett’s esophagus
EAC: Esophageal adenocarcinoma
GEO: Gene Expression Omnibus

## References

[1] Pace E, Di Vincenzo S, Ferraro M, Bruno A, Dino P, Bonsignore MR, et al. Carbocysteine counteracts the effects of cigarette smoke on cell growth and on the SIRT1/FoxO3 axis in bronchial epithelial cells. Exp Gerontol 2016;81:119-128. PubMed PMID: 27237816. 10.1016/j.exger.2016.05.013 27237816

[2] Walters MS, De BP, Salit J, Buro-Auriemma LJ, Wilson T, Rogalski AM (2014). Smoking accelerates aging of the small airway epithelium. Respir Res.

[3] Nicita-Mauro V, Lo Balbo C, Mento A, Nicita-Mauro C, Maltese G, Basile G. Smoking, aging and the centenarians. Exp Gerontol 2008;43(2):95-101. PubMed PMID: 17686596. 10.1016/j.exger.2007.06.011 17686596

[4] Liu Y, Pleasants RA, Croft JB, Wheaton AG, Heidari K, Malarcher AM, et al. Smoking duration, respiratory symptoms, and COPD in adults aged >/=45 years with a smoking history. Int J Chron Obstruct Pulmon Dis 2015;10:1409-1416. PubMed PMID: 26229460. Pubmed Central PMCID: 4516194. 10.2147/COPD.S82259 PMC4516194 26229460

[5] Nicita-Mauro V, Maltese G, Nicita-Mauro C, Lasco A, Basile G (2010). Non smoking for successful aging: therapeutic perspectives. Curr Pharm Des.

[6] Chien AL, Qi J, Cheng N, Do TT, Mesfin M, Egbers R, et al. Perioral wrinkles are associated with female gender, aging, and smoking: development of a gender-specific photonumeric scale. J Am Acad Dermatol. 2016;74(5):924-930. PubMed PMID: 26803346. 10.1016/j.jaad.2015.11.042 26803346

[7] Vij N, Chandramani-Shivalingappa P, Van Westphal C, Hole R, Bodas M. Cigarette smoke-induced autophagy impairment accelerates lung aging, COPD-emphysema exacerbations and pathogenesis. Am J Physiol Cell Physiol. 2018;314(1):C73-C87. PubMed PMID: 27413169. Pubmed Central PMCID: 5866380. 10.1152/ajpcell.00110.2016 PMC5866380 27413169

[8] Gao X, Zhang Y, Breitling LP, Brenner H. Tobacco smoking and methylation of genes related to lung cancer development. Oncotarget. 2016;7(37):59017-59028. PubMed PMID: 27323854. eng. 10.18632/oncotarget.10007 PMC5312292 27323854

[9] Willemse BWM, Postma DS, Timens W, ten Hacken NHT (2004). The impact of smoking cessation on respiratory symptoms, lung function, airway hyperresponsiveness and inflammation. Eur Respir J.

[10] Tsaprouni LG, Yang TP, Bell J, Dick KJ, Kanoni S, Nisbet J, et al. Cigarette smoking reduces DNA methylation levels at multiple genomic loci but the effect is partially reversible upon cessation. Epigenetics 2014;9(10):1382-1396. PubMed PMID: 25424692. Pubmed Central PMCID: 4623553. 10.4161/15592294.2014.969637 PMC4623553 25424692

[11] Hannum G, Guinney J, Zhao L, Zhang L, Hughes G, Sadda S (2013). Genome-wide Methylation Profiles Reveal Quantitative Views of Human Aging Rates. Mol Cell..

[12] Horvath S. DNA methylation age of human tissues and cell types. Genome Biol. 2013;14(10):R115-R11R. PubMed PMID: 24138928. Epub 10/21. 10.1186/gb-2013-14-10-r115 PMC4015143 24138928

[13] Levine ME, Lu AT, Quach A, Chen BH, Assimes TL, Bandinelli S, et al. An epigenetic biomarker of aging for lifespan and healthspan. Aging (Milano). 2018;10(4):573-591. PubMed PMID: 29676998. eng. 10.18632/aging.101414 PMC5940111 29676998

[14] Ambaipudi S, Cuenin C, Hernandezvargas H, Ghantous A, Calvezkelm FL, Kaaks R (2016). Tobacco smoking-associated genome-wide DNA methylation changes in the EPIC study. Epigenomics.

[15] Quach A, Levine ME, Tanaka T, Lu AT, Chen BH, Ferrucci L, et al. Epigenetic clock analysis of diet, exercise, education, and lifestyle factors. Aging (Milano) 2017;9(2):419-446. PubMed PMID: 28198702. 10.18632/aging.101168 PMC5361673 28198702

[16] Horvath S, Erhart W, Brosch M, Ammerpohl O, von Schönfels W, Ahrens M, et al. Obesity accelerates epigenetic aging of human liver. Proc Natl Acad Sci U S A. 2014;111(43):15538-15543. PubMed PMID: 25313081. Epub 10/13. 10.1073/pnas.1412759111 PMC4217403 25313081

[17] Zannas AS, Arloth J, Carrillo-Roa T, Iurato S, Röh S, Ressler KJ, et al. Lifetime stress accelerates epigenetic aging in an urban, African American cohort: relevance of glucocorticoid signaling. Genome Biol. 2015;16:266. PubMed PMID: 26673150. 10.1186/s13059-015-0828-5 PMC4699359 26673150

[18] Horvath S, Ritz BR. Increased epigenetic age and granulocyte counts in the blood of Parkinson's disease patients. Aging (Milano) 2015;7(12):1130-1142. PubMed PMID: 26655927. 10.18632/aging.100859 PMC4712337 26655927

[19] Horvath S, Garagnani P, Bacalini MG, Pirazzini C, Salvioli S, Gentilini D, et al. Accelerated epigenetic aging in Down syndrome. Aging cell. 2015;14(3):491-495. PubMed PMID: 25678027. Epub 02/09. 10.1111/acel.12325 PMC4406678 25678027

[20] Vidal-Bralo L, Lopez-Golan Y, Mera-Varela A, Rego-Perez I, Horvath S, Zhang Y, et al. Specific premature epigenetic aging of cartilage in osteoarthritis. Aging (Milano) 2016;8(9):2222-2231. PubMed PMID: 27689435. 10.18632/aging.101053 PMC5076459 27689435

[21] Levine ME, Lu AT, Bennett DA, Horvath S. Epigenetic age of the pre-frontal cortex is associated with neuritic plaques, amyloid load, and Alzheimer's disease related cognitive functioning. Aging (Milano) 2015;7(12):1198-1211. PubMed PMID: 26684672. 10.18632/aging.100864 PMC4712342 26684672

[22] Hsu CC, Leu YW, Tseng MJ, Lee KD, Kuo TY, Yen JY, et al. Functional characterization of Trip10 in cancer cell growth and survival. J Biomed Sci. 2011;18:12. PubMed PMID: 21299869. Pubmed Central PMCID: 3044094. 10.1186/1423-0127-18-12 PMC3044094 21299869

[23] McLoughlin KJ, Pedrini E, MacMahon M, Guduric-Fuchs J, Medina RJ. Selection of a Real-Time PCR Housekeeping Gene Panel in Human Endothelial Colony Forming Cells for Cellular Senescence Studies. Front Med (Lausanne). 2019;6. 10.3389/fmed.2019.00033 PMC6421261 30915334

[24] Donlon TA, Morris BJ, Chen R, Masaki KH, Allsopp RC, Willcox DC, et al. Analysis of Polymorphisms in 59 Potential Candidate Genes for Association With Human Longevity. J Gerontol A Biol Sci Med Sci 2018;73(11):1459-1464. PubMed PMID: 29300832. Pubmed Central PMCID: 6175033. 10.1093/gerona/glx247 PMC6175033 29300832

[25] Di Donato N, Jean YY, Maga AM, Krewson BD, Shupp AB, Avrutsky MI, et al. Mutations in CRADD Result in Reduced Caspase-2-Mediated Neuronal Apoptosis and Cause Megalencephaly with a Rare Lissencephaly Variant. Am J Hum Genet. 2016;99(5):1117-1129. PubMed PMID: 27773430. Pubmed Central PMCID: 5097945. 10.1016/j.ajhg.2016.09.010 PMC5097945 27773430

[26] Li Y, Xiao X, Ji X, Liu B, Amos CI. RNA-seq analysis of lung adenocarcinomas reveals different gene expression profiles between smoking and nonsmoking patients. Tumour Biol 2015 Nov;36(11):8993-9003. PubMed PMID: 26081616. Pubmed Central PMCID: 4674426. 10.1007/s13277-015-3576-y PMC4674426 26081616

[27] Rapisarda V, Borghesan M, Miguela V, Encheva V, Snijders AP, Lujambio A, et al. Integrin Beta 3 Regulates Cellular Senescence by Activating the TGF-beta Pathway. Cell Rep 2017;18(10):2480-2493. PubMed PMID: 28273461. Pubmed Central PMCID: 5357738. 10.1016/j.celrep.2017.02.012 PMC5357738 28273461

[28] Yin J, Yang L, Xie Y, Liu Y, Li S, Yang W, et al. Dkk3 dependent transcriptional regulation controls age related skeletal muscle atrophy. Nat Commun 2018;9(1):1752. PubMed PMID: 29717119. Pubmed Central PMCID: 5931527. 10.1038/s41467-018-04038-6 PMC5931527 29717119

[29] Bruneau BG, Nemer G, Schmitt JP, Charron F, Robitaille L, Caron S (2001). A Murine Model of Holt-Oram Syndrome Defines Roles of the T-Box Transcription Factor Tbx5 in Cardiogenesis and Disease. Cell.

[30] Lengi AJ, Corl BA. Bovine brain region-specific stearoyl-CoA desaturase expression and fatty acid composition. Lipids 2015 Jun;50(6):555-563. PubMed PMID: 25899038. 10.1007/s11745-015-4015-y 25899038

[31] Geng RQ, Yuan C, Chen YL. Molecular cloning and expression analysis of prostaglandin E receptor 2 gene in cashmere goat (Capra hircus) skin during hair follicle development. Anim Biotechnol 2014;25(2):98-107. PubMed PMID: 24555795. 10.1080/10495398.2013.826236 24555795

[32] Prall WC, Czibere A, Jager M, Spentzos D, Libermann TA, Gattermann N, et al. Age-related transcription levels of KU70, MGST1 and BIK in CD34+ hematopoietic stem and progenitor cells. Mech Ageing Dev 2007;128(9):503-510. PubMed PMID: 17714764. 10.1016/j.mad.2007.06.008 17714764

[33] Rigotti NA (2013). Smoking cessation in patients with respiratory disease: existing treatments and future directions. Lancet Respir Med.

[34] Philibert R, Hollenbeck N, Andersen E, McElroy S, Wilson S, Vercande K, et al. Reversion of AHRR Demethylation Is a Quantitative Biomarker of Smoking Cessation. Front Psychiatry 2016;7:55. PubMed PMID: 27092088. Pubmed Central PMCID: 4822186. 10.3389/fpsyt.2016.00055 PMC4822186 27092088

[35] Langevin SM, Eliot M, Butler RA, Cheong A, Zhang X, McClean MD, et al. CpG island methylation profile in non-invasive oral rinse samples is predictive of oral and pharyngeal carcinoma. Clin Epigenetics. 2015;7:125. PubMed PMID: 26635906. Pubmed Central PMCID: 4668652. 10.1186/s13148-015-0160-7 PMC4668652 26635906

[36] Jessen WJ, Borgerding MF, Prasad GL. Global methylation profiles in buccal cells of long-term smokers and moist snuff consumers. Biomarkers. 2018 Nov;23(7):625-639. PubMed PMID: 29771158. 10.1080/1354750X.2018.1466367 29771158

[37] Vucic EA, Chari R, Thu KL, Wilson IM, Cotton AM, Kennett JY, et al. DNA methylation is globally disrupted and associated with expression changes in chronic obstructive pulmonary disease small airways. Am J Respir Cell Mol Biol 2014;50(5):912-922. PubMed PMID: 24298892. Pubmed Central PMCID: 4068945. 10.1165/rcmb.2013-0304OC PMC4068945 24298892

[38] Clifford RL, Fishbane N, Patel J, MacIsaac JL, McEwen LM, Fisher AJ, et al. Altered DNA methylation is associated with aberrant gene expression in parenchymal but not airway fibroblasts isolated from individuals with COPD. Clin Epigenetics 2018;10:32. PubMed PMID: 29527240. Pubmed Central PMCID: 5838860. 10.1186/s13148-018-0464-5 PMC5838860 29527240

[39] Kaz AM, Wong CJ, Varadan V, Willis JE, Chak A, Grady WM. Global DNA methylation patterns in Barrett's esophagus, dysplastic Barrett's, and esophageal adenocarcinoma are associated with BMI, gender, and tobacco use. Clin Epigenetics 2016;8:111. PubMed PMID: 27795744. Pubmed Central PMCID: 5082363. 10.1186/s13148-016-0273-7 PMC5082363 27795744

[40] Sundar IK, Yin Q, Baier BS, Yan L, Mazur W, Li D, et al. DNA methylation profiling in peripheral lung tissues of smokers and patients with COPD. Clin Epigenetics 2017;9:38. PubMed PMID: 28416970. Pubmed Central PMCID: 5391602. 10.1186/s13148-017-0335-5 PMC5391602 28416970

[41] Robles AI, Arai E, Mathe EA, Okayama H, Schetter AJ, Brown D, et al. An Integrated Prognostic Classifier for Stage I Lung Adenocarcinoma Based on mRNA, microRNA, and DNA Methylation Biomarkers. J Thorac Oncol 2015;10(7):1037-1048. PubMed PMID: 26134223. Pubmed Central PMCID: 4493931. 10.1097/JTO.0000000000000560 PMC4493931 26134223

[42] Kajiura K, Masuda K, Naruto T, Kohmoto T, Watabnabe M, Tsuboi M, et al. Frequent silencing of the candidate tumor suppressor TRIM58 by promoter methylation in early-stage lung adenocarcinoma. Oncotarget. 2016;8(2):2890-2905. PubMed PMID: 27926516. eng. 10.18632/oncotarget.13761 PMC5356850 27926516

[43] Kettunen E, Hernandez-Vargas H, Cros MP, Durand G, Le Calvez-Kelm F, Stuopelyte K, et al. Asbestos-associated genome-wide DNA methylation changes in lung cancer. Int J Cancer. 2017;141(10):2014-2029. PubMed PMID: 28722770. 10.1002/ijc.30897 28722770

