# Supplementary material for: Effect of tobacco smoking on the epigenetic age of human respiratory organs
Source: Clin Epigenetics. 2019 Dec 4;11:183. doi: 10.1186/s13148-019-0777-z (PMC6894291; doi:10.1186/s13148-019-0777-z)
Supplement: Supplementary file 1 — Additional file 1: Table S1. Summary of the DNA methylation datasets. [file 13148_2019_777_MOESM1_ESM.docx]

**Supplement Table 1: Summary of the DNA methylation datasets**

| ID | Tissue source | Availability (GEO ID) | Platform | Number of total | Number of Female | Mean Age (range) | Citation |
| --- | --- | --- | --- | --- | --- | --- | --- |
| 1 | Oral Rinse | GSE70977 | 450K | 223 | 70 | 60 (23-88) | Langevin et al.[39] |
| 2 | Buccal Cells | GSE94876 | 450K | 120 | NA | 46 (35-60) | Jessen et al.[40] |
| 3 | Small Airway Epithelial Cells | GSE55454 | 27K | 38 | 13 | 64 (51-75) | Vucic et al.[41] |
| 4 | Airway Fibroblasts | GSE111396 | 450K | 61 | 26 | 65 (43-83) | Clifford et al.[42] |
| 5 | Esophagus Tissues | GSE89181 | 450K | 127 | 14 | 67 (40-93) | Kaz et al.[43] |
| 6 | Lung Tissues | GSE92511 | 450K | 24 | 6 | 60 (45-71) | Sundar et al.[44] |
| 7 | Lung Tissues | GSE63384 | 27K | 70 | 38 | 65 (47-88) | Robles et al.[45] |
| 8 | Lung Tissues | GSE83842 | 450K | 24 | 0 | 67 (51-80) | Kajiura et al.[46] |
| 9 | Lung Tissues | GSE94785 | 450K | 62 | 0 | 61 (32-78) | Kettunen et al.[47] |
